# Multiplexed In Situ Protein Profiling with High-Performance Cleavable Fluorescent Tyramide

**DOI:** 10.3390/molecules26082206

**Published:** 2021-04-12

**Authors:** Thai Pham, Renjie Liao, Joshua Labaer, Jia Guo

**Affiliations:** Biodesign Institute & School of Molecular Sciences, Arizona State University, Tempe, AZ 85287, USA; thpham7@asu.edu (T.P.); Renjie.Liao@asu.edu (R.L.); Joshua.Labaer@asu.edu (J.L.)

**Keywords:** single-cell, proteomics, immunofluorescence, immunohistochemistry, heterogeneity

## Abstract

Understanding the composition, function and regulation of complex cellular systems requires tools that quantify the expression of multiple proteins at their native cellular context. Here, we report a highly sensitive and accurate protein in situ profiling approach using off-the-shelf antibodies and cleavable fluorescent tyramide (CFT). In each cycle of this method, protein targets are stained with horseradish peroxidase (HRP) conjugated antibodies and CFT. Subsequently, the fluorophores are efficiently cleaved by mild chemical reagents, which simultaneously deactivate HRP. Through reiterative cycles of protein staining, fluorescence imaging, fluorophore cleavage, and HRP deactivation, multiplexed protein quantification in single cells in situ can be achieved. We designed and synthesized the high-performance CFT, and demonstrated that over 95% of the staining signals can be erased by mild chemical reagents while preserving the integrity of the epitopes on protein targets. Applying this method, we explored the protein expression heterogeneity and correlation in a group of genetically identical cells. With the high signal removal efficiency, this approach also enables us to accurately profile proteins in formalin-fixed paraffin-embedded (FFPE) tissues in the order of low to high and also high to low expression levels.

## 1. Introduction

Highly multiplexed protein profiling in their native spatial contexts holds great promise for revealing the composition, regulation and interaction of the various cell types in complex cellular systems [1,2]. Protein microarray [3] and mass spectrometry [4] are well-established methods for proteomic analysis. However, as these approaches do not quantify proteins in their original cellular environment, the location information of the proteins is lost during analysis. Immunofluorescence is a powerful tool for in situ protein quantification. Nonetheless, due to the spectral overlap of the common fluorophores, immunofluorescence only allows a small number of varied proteins to be profiled on each specimen [5].

To allow multiplexed in situ protein analysis, several techniques [6,7,8,9,10,11,12,13,14] have been explored. In these approaches, the fluorophores or metal isotopes directly conjugated antibodies are applied as detection tags. Without further signal amplification, these approaches suffer from low detection sensitivity, which limits their application for the analysis of low expression proteins or the studying of highly autofluorescent specimens, such as formalin fixed, paraffin embedded (FFPE) tissues [7]. Recently, some signal amplification methods have been developed for multiplexed protein imaging [15,16]. However, these approaches require a chemical or oligonucleotide tag to be conjugated to primary antibodies. Preparing those tag conjugated primary antibodies can be time-consuming and expensive. More importantly, the bulky chemical or oligonucleotide tag can interfere with the binding affinity and specificity of the primary antibodies.

To enable highly sensitive and multiplexed protein imaging with off-the-shelf antibodies, our group developed a reiterative protein staining approach using cleavable fluorescent tyramide (CFT) [17]. We demonstrated that its sensitivity is improved by about two orders of magnitude compared with other existing methods. As a result, its imaging time is dramatically reduced, and the sample throughput is significantly enhanced. However, some nonideal factors still exist. For example, the carbamate group in the first-generation CFT could potentially react with the nucleophiles in the cellular environment or during storage, which may lead to side reactions or short shelf life. Additionally, with tris(2-carboxyethyl) phosphine (TCEP) as the signal removal reagent, the first-generation CFT requires 65 °C to remove ~95% of the staining signals. Therefore, this relatively high reaction temperature could damage the integrity of the epitopes [17].

Here, we report a highly sensitive and multiplexed in situ protein analysis method using high-performance CFT. In this approach, protein targets are recognized by antibodies conjugated with horseradish peroxidase (HRP) and then stained with CFT. Without the carbamate group in this newly designed CFT, it avoids the potential side reactions with the cellular nucleophiles. Additionally, over 95% of the staining signals can be efficiently removed using 1,3,5-Triaza-7-phosphaadamantane (PTA) and TCEP at 40° C. Simultaneously, HRP is also effectively deactivated under this mild condition. Through reiterative cycles of target staining, fluorescence imaging, signal erasing and HRP quenching, we demonstrated at least 10 reiterative immunofluorescence cycles can be successfully carried out in cultured cells. With the generated multiplexed single-cell in situ protein profiling data, we explored the protein expression heterogeneity and correlation in a population of genetically identical cells. We also showed that the significantly improved signal removal efficiency of our approach enables the accurate quantification of multiple proteins in the order of low to high and also high to low expression levels in FFPE tissues.

## 2. Results

### 2.1. Platform Design

**Figure 1:**
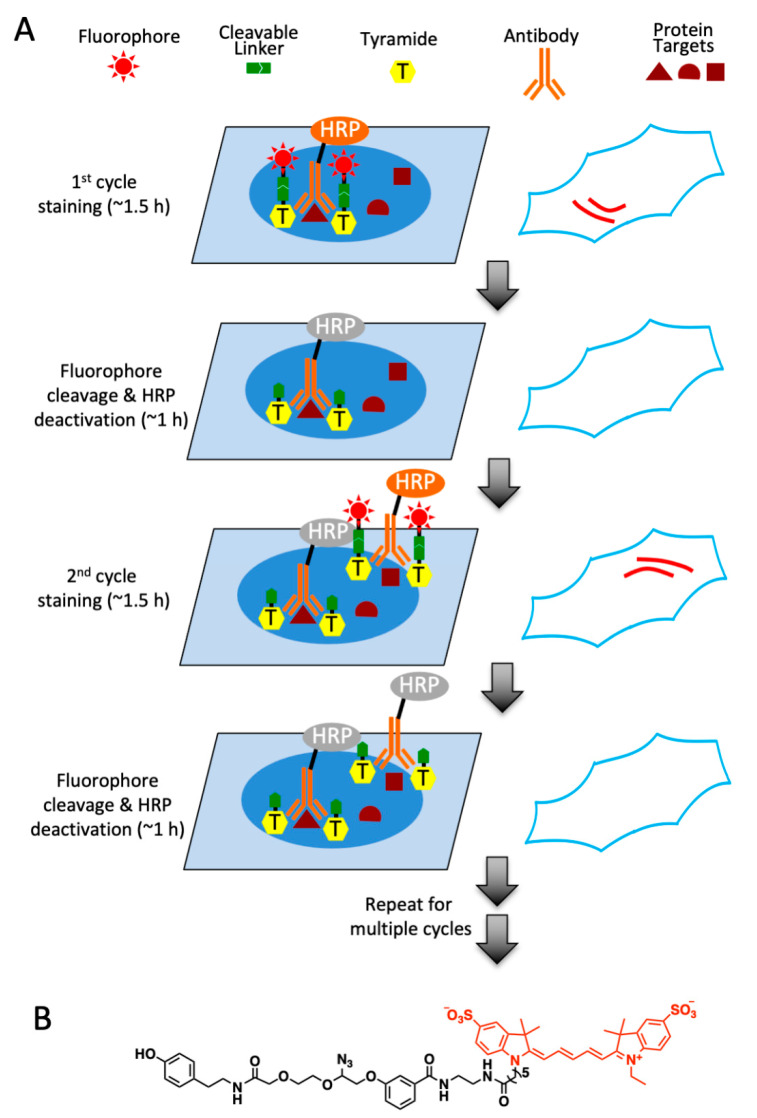
(**A**) Multiplexed in situ protein profiling with high-performance cleavable fluorescent tyramide. After staining protein targets with horseradish peroxidase (HRP) conjugated antibodies and cleavable fluorescent tyramide, images are captured for protein profiling. Subsequently, a mild chemical reaction is applied to cleave the fluorophores and deactivate HRP simultaneously. Upon reiterative cycles of protein staining, imaging, fluorophore removal and HRP deactivation, multiplexed protein analysis can be achieved in individual cells in situ. (**B**) Chemical structure of high-performance cleavable fluorescent tyramide, tyramide-N_3_-Cy5.

In this multiplexed protein imaging approach, each staining cycle was composed of three major steps (Figure 1A). First, the protein of interest was recognized by off-the-shelf antibodies labeled with HRP, which catalyzed the conversion of the tyramide moiety in CFT into a highly reactive radical. This radical is short-lived, and only covalently binds to the tyrosine residues on the proteins proximal to the antibodies. Second, the specimen was imaged under a fluorescence microscope to generate quantitative single-cell in situ protein expression profiles. The nucleus stained with DAPI could be imaged in each cycle as a reference to facilitate the image alignment and overlay in different cycles. In the last step, the fluorophores tethered to tyramide were efficiently removed by chemical cleavage, and HRP was simultaneously deactivated. With continuous cycles of staining, imaging, cleavage and HRP deactivation, highly multiplexed and sensitive protein profiling can be achieved in single cells in situ.

### 2.2. Design and Synthesis of High Performance CFT

To eliminate the potential side reactions with the cellular nucleophiles and also improve the shelf life, the high-performance CFT should avoid the carbamate group. Additionally, the linker tethering the fluorophore and tyramide must be cleaved efficiently under a mild condition by a bioorthogonal reaction. Our group has recently developed a cleavable fluorescent oligonucleotide for comprehensive RNA and DNA in situ profiling [18]. The azide-based linker used in that method satisfies the two requirements for successful CFT. Therefore, that linker was applied to couple the fluorophore to tyramide in high performance CFT. Most tissues exhibit stronger autofluorescence in the green and yellow emission channels than in the red channel [19]. Thus, to minimize the impact of autofluorescence on the accurate protein analysis, we applied Cy5 as the fluorophore in CFT under the current study.

To synthesize the designed tyramide-N_3_-Cy5 (Figure 1B), Cy5 NHS ester was first coupled to the azide-based linker, as previously reported in the literature. The generated product was subsequently converted into the NHS ester and conjugated with tyramine. The prepared CFT was purified by high performance liquid chromatography (HPLC) and characterized by nuclear magnetic resonance (NMR) spectroscopy and mass spectrometry.

### 2.3. Efficient Fluorophore Cleavage While Preserving Epitope Integrity

**Figure 2:**
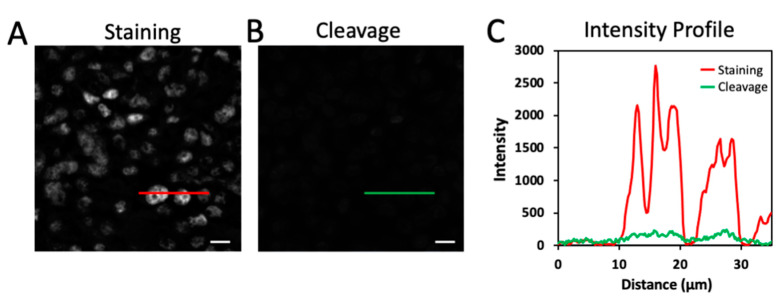
(**A**) Protein ILF3 in a human tonsil FFPE tissue is stained with tyramide-N_3_-Cy5. (**B**) The fluorescence signal is removed by PTA and TCEP. (**C**) Fluorescence intensity profile corresponding to the red and green line positions in (**A**) and (**B**). Scale bars, 10 μm.

One of the critical requirements for the success of the multiplexed protein imaging approach was to efficiently remove the staining signals without loss of protein antigenicity. In this way, the signal leftover generated in the previous cycles could be minimized, and other protein targets could still be successfully recognized by antibodies in the following immunofluorescence cycles. To assess the fluorophore cleavage efficiency of the high-performance CFT, we stained protein ILF3 in a human tonsil FFPE tissue (Figure 2A). To facilitate the efficient signal removal in different microenvironment, we applied the positively charged phosphine PTA in combination with the negatively charged phosphine TCEP to perform fluorophore cleavage. After incubating with PTA and TCEP at 40 °C for 30 min, over 95% of the signals were removed (Figure 2B,C). Compared with the first-generation CFT, the reaction temperature of 65 °C is required to achieve the similar cleavage efficiency. However, that high cleavage temperature leads to the partial loss of protein antigenicity. The results here suggest that the fluorophore in the high-performance CFT can be very efficiently removed under a relatively low cleavage temperature.

**Figure 3:**
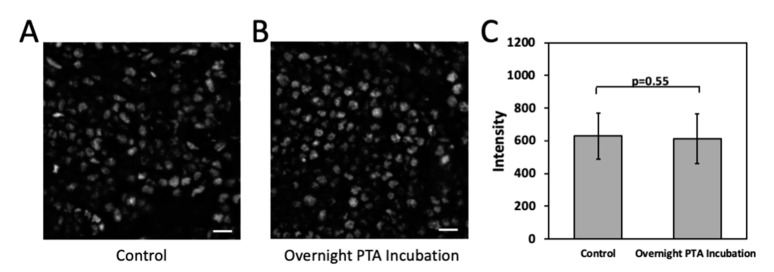
(**A**) Protein ILF3 in in a human tonsil FFPE tissue is directly stained with tyramide-N_3_-Cy5. (**B**) After the overnight PTA incubation, Protein ILF3 in in a human tonsil FFPE tissue is stained with tyramide-N_3_-Cy5. (**C**) Fluorescence intensity of ILF3 staining in (**A**,**B**) (n = 30 positions). Error bars generated using standard error of the mean. Scale bars, 10 μm.

We next investigated whether the epitope integrity is preserved under this cleavage condition. Previously, we have shown that the TCEP treatment at this relatively low temperature does not result in loss of protein antigenicity [8]. Thus, here we evaluated the effects of the PTA incubation on the epitope integrity (Figure 3). After the PTA treatment overnight, protein ILF3 was stained in a human tonsil FFPE tissue. The generated staining patterns (Figure 3A,B) and signal intensities (Figure 3C) are consistent with the ones obtained without the PTA treatment. These results indicate that the antibody antigenicity is maintained under our mild fluorophore cleavage condition.

### 2.4. Simultaneous HRP Deactivation and Fluorophore Cleavage

**Figure 4:**
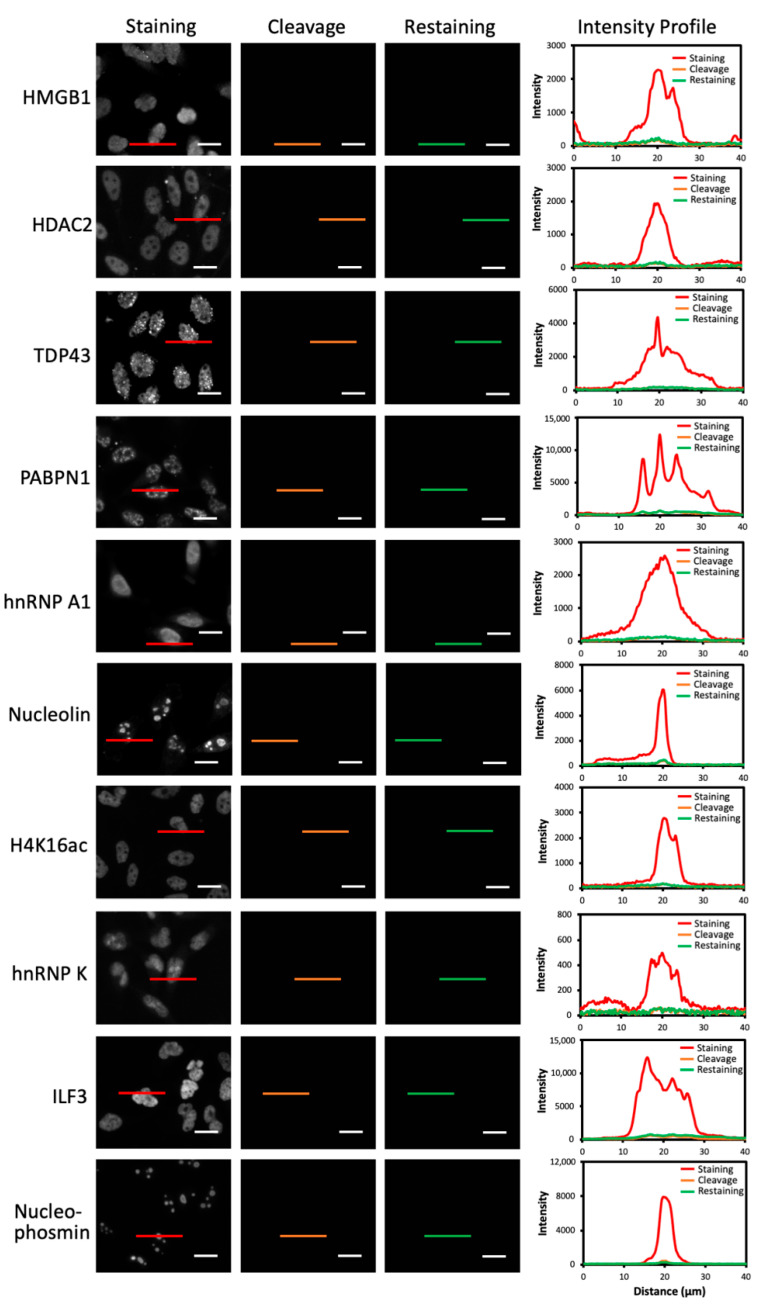
Ten proteins in HeLa cells were stained with tyramide-N_3_-Cy5 (the first column). Subsequently, the staining signals were removed by PTA and TCEP (the second column). Then, the cells were reincubated with tyramide-N_3_-Cy5 (the third column). The fourth column shows the signal intensity profiles corresponding to the red, orange and green line positions in the first three columns. Scale bars, 15 μm.

To enable accurate protein expression analysis by this reiterative imaging approach, it also requires the HRP on antibodies to be deactivated after the staining images have been captured in each analysis cycle. In this way, the HRP applied in the previous cycles will not produce false positive signals in the following cycles. To evaluate whether PTA and TCEP can cleave the fluorophore and deactivate HRP simultaneously, we stained HMGB1, HDAC2, TAP43, PABPN1, hnRNP A1, Nucleolin, H4K16ac, hnRNP K, ILF3 and Nucleophosmin with HRP labeled antibodies and the high-performance tyramide-N_3_-Cy5 in HeLa cells (Figure 4). All the antibodies used in this study are well-validated and documented in the literature [20,21,22,23,24,25,26,27,28,29]. With the PTA and TCEP treatment, over 95% of the staining signals were erased, leading to the on/off ratio of about 20:1. After signal removal, we stained the cells again with tyramide-N_3_-Cy5. No signal increases were observed for any of the 10 proteins. These results confirmed that PTA and TCEP could efficiently cleave the fluorophores in the high-performance CFT, and also suggested that the HRP was almost completely deactivated simultaneously.

### 2.5. Multiplexed Protein Imaging in Cultured Cells

**Figure 5:**
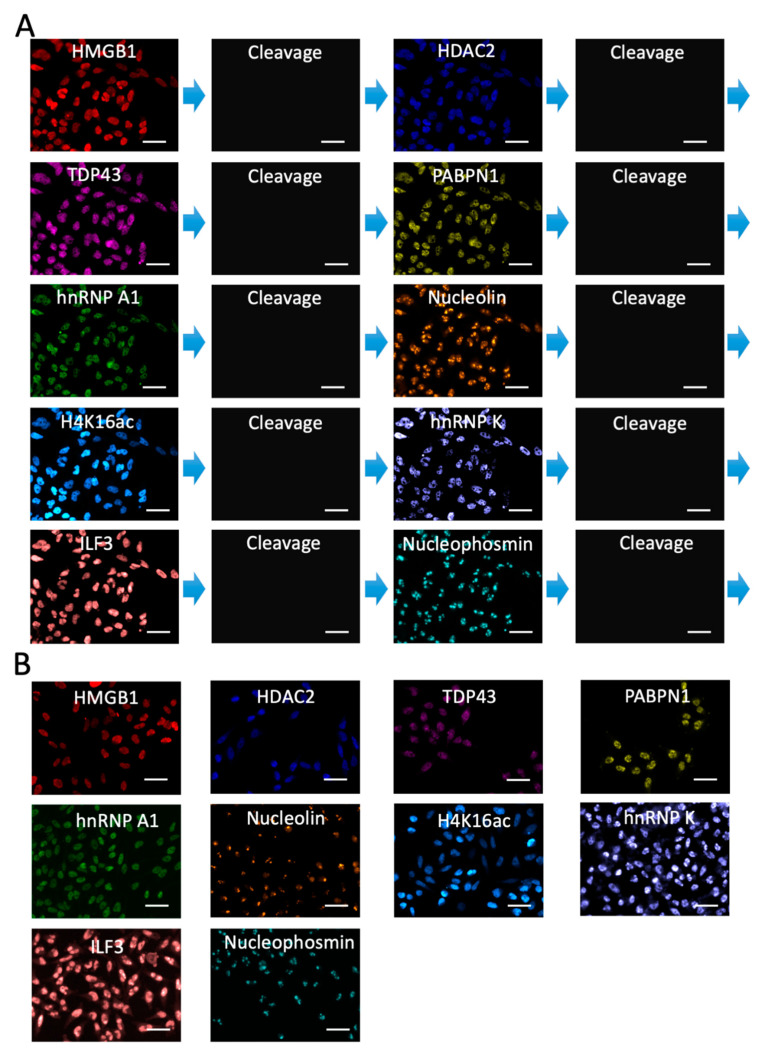
(**A**) Ten proteins were sequentially stained in the same set of HeLa cells with tyramide-N_3_-Cy5. (**B**) Ten proteins were individually stained in different HeLa cells with conventional TSA. Scale bars, 50 μm.

**Figure 6:**
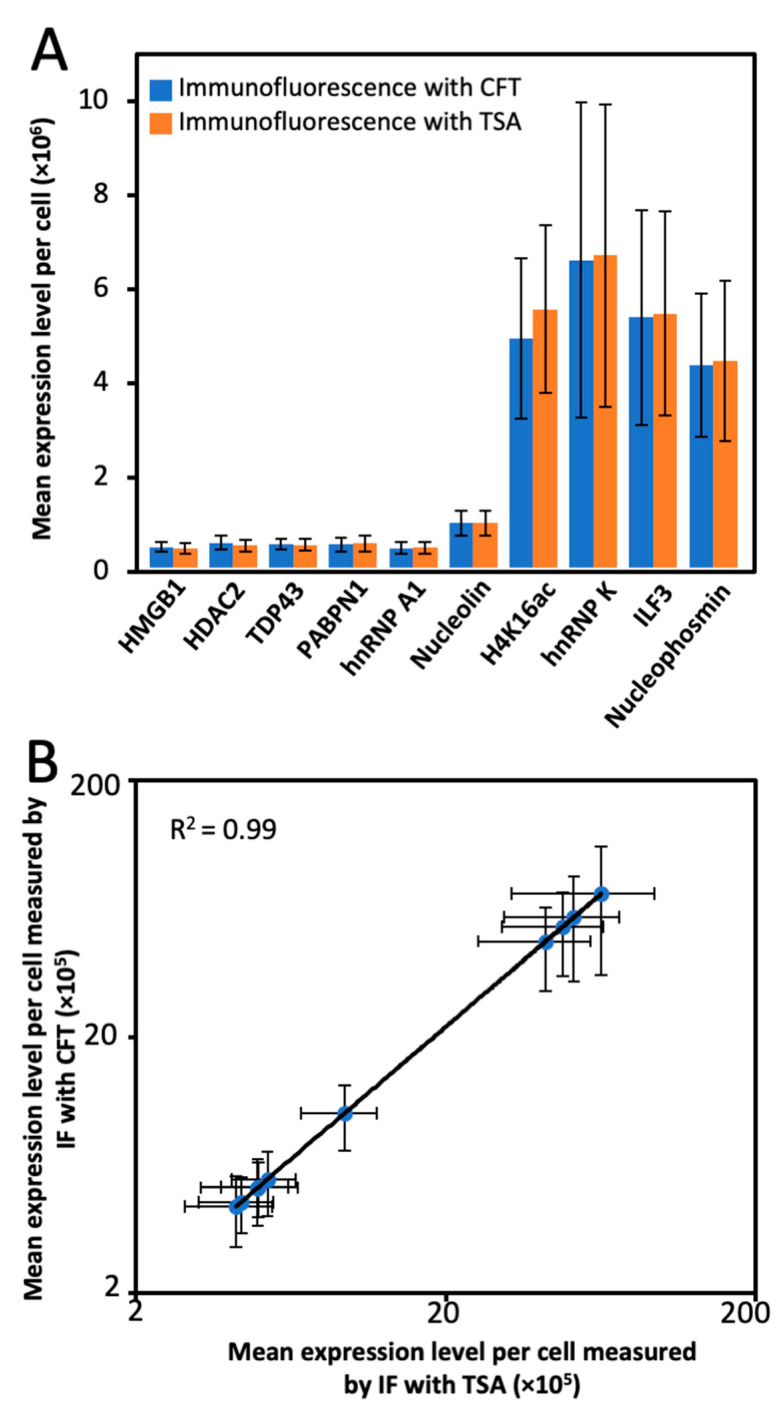
(**A**) Mean expression levels per cell (n = 100 cells) of the 10 proteins quantified by tyramide-N_3_-Cy5 and conventional TSA. (**B**) Comparison of the results obtained by tyramide-N_3_-Cy5 and TSA yields R^2^ = 0.99 with a slope of 1.09. Error bars generated using standard error of the mean.

To explore whether the high-performance CFT could be applied for multiplexed protein imaging, we stained the 10 different proteins through reiterative immunofluorescence cycles in the same set of HeLa cells (Figure 5A). As controls, we also stained each of those 10 target proteins using the conventional tyramide signal amplification (TSA) approach (Figure 5B). The protein distribution patterns generated by the two approaches, together with the ones obtained with antibodies from varied clones [30,31], closely resembled each other. Additionally, the mean protein expression levels per cell obtained using our and TSA methods were also consistent (Figure 6A). Comparison of the results measured by the two approaches generated an R^2^ = 0.99 with a slope of 1.09 (Figure 6B). These results suggest that our method enables the accurate and multiplexed protein analysis in single cells in situ.

### 2.6. Single-Cell Protein Expression Heterogeneity and Correlation

**Figure 7:**
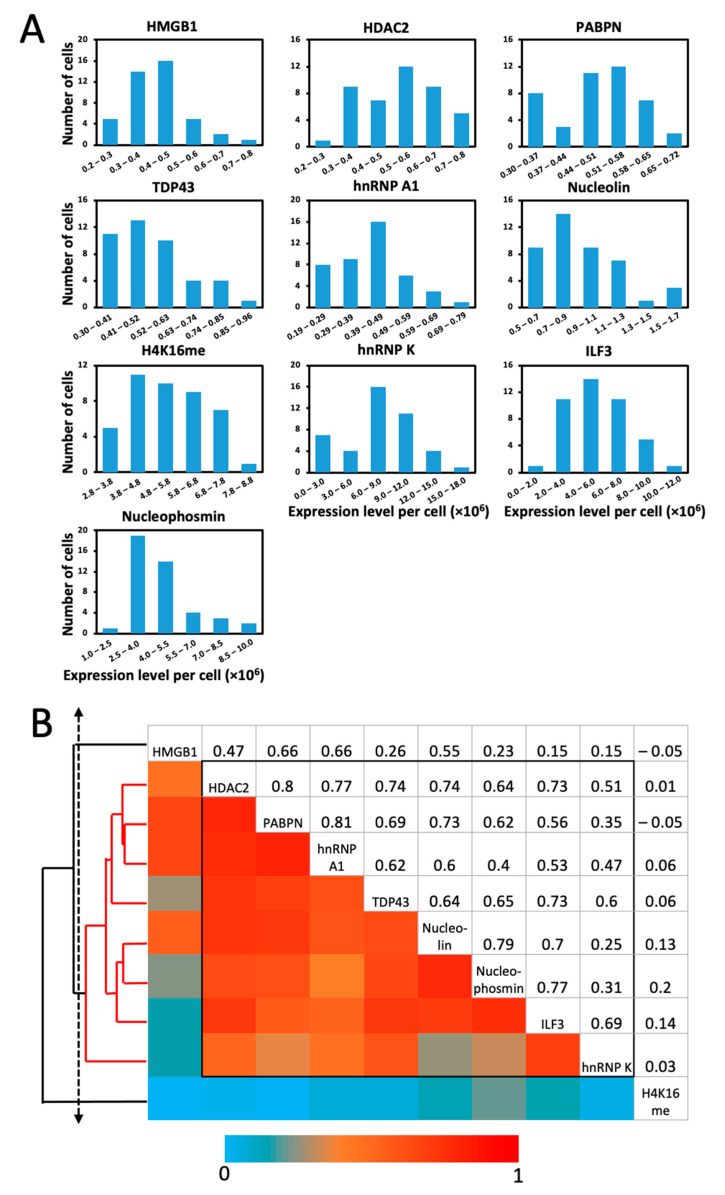
(**A**) Histograms of the expression levels per cell for the 10 proteins stained in HeLa cells. (**B**) Pair-wise correlation of the expression levels of the 10 proteins and the hierarchical clustering tree. In the upper triangle, the expression correlation coefficients of every protein pair are shown. In the lower triangle, the color corresponds to the correlation coefficients of each protein pair. The diagonal displays the names of the proteins. With a threshold on the cluster tree (dashed line), a group of proteins with high expression correlation are identified and indicated by the red lines on the clustering tree and the black box in the matrix.

As reported previously, a group of genetically identical cells can have varied gene expression patterns at the single cell level [32,33,34,35,36,37,38]. To study such protein expression heterogeneity in individual HeLa cells, we quantified the distribution of the protein abundances in single cells (Figure 7A). The expression levels of all the 10 analyzed proteins were distributed widely, which led to the relatively large expression standard deviations in Figure 6. The square of those expression standard deviations was significantly higher than the average expression levels for each measured protein. These results suggest that the protein expression in individual HeLa cells is highly heterogeneous, which may be attributed to the synthesis of those proteins in bursts [39].

To explore which proteins are coregulated in a regulatory pathway, one can perform protein expression covariation analysis. Such studies carried out in populations of cells usually require external stimuli to induce different protein expression levels in varied cell groups. With the gene expression heterogeneity naturally generated in individual cells, single-cell protein expression covariation analysis can advance our understanding of regulatory pathways and predict the potential function of unannotated proteins [40]. To perform such studies, we calculated the pairwise expression correlation coefficient of the 10 analyzed proteins. Some protein pairs exhibited high expression correlation with the correlation efficient of ~0.8, including HDAC2 and PABPN together with PABPN and hnRNP A1. To elucidate the regulatory network among the 10 analyzed proteins, a hierarchical clustering method [41] was used and identified a group of eight proteins with substantial expression correlation (Figure 7B). As reported in the literature [42,43,44,45,46,47,48,49], those eight proteins are indeed involved in the transcription processing and regulation pathways.

### 2.7. Multiplexed In Situ Protien Profiling in FFPE Tissues

**Figure 8:**
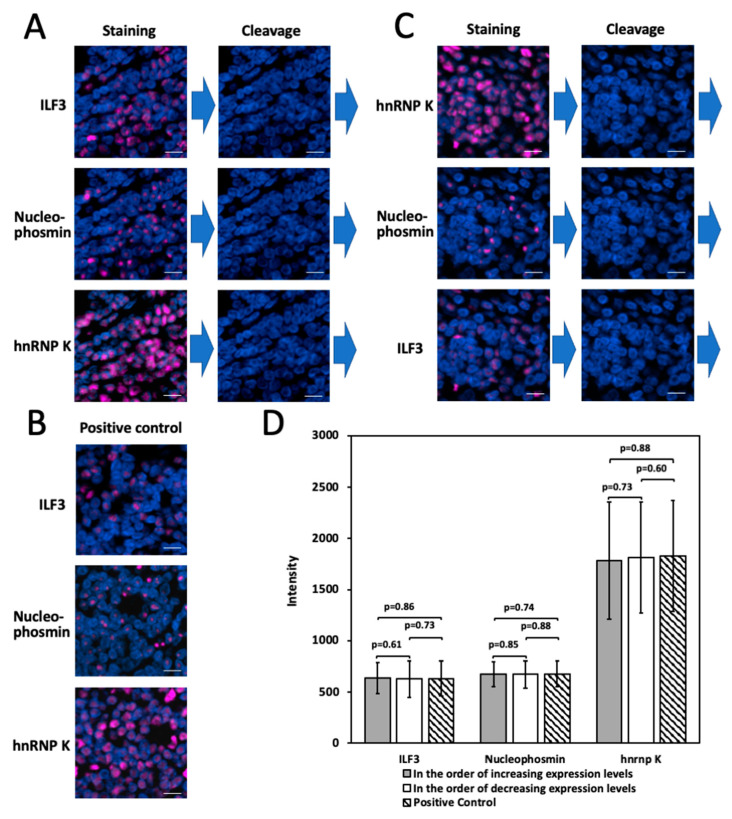
(**A**) In the order of increasing expression levels, three proteins were sequentially stained in a human tonsil FFPE tissue. (**B**) The three proteins were stained by conventional TSA in three different human tonsil FFPE tissues. (**C**) In the order of decreasing expression levels, the same three proteins were sequentially stained in a human tonsil FFPE tissue. (**D**) Fluorescence intensity of the three proteins stained in (**A**–**C**) (n = 50 positions). Error bars generated using standard error of the mean. Scale bars, 10 μm.

To demonstrate the feasibility of applying the high performance CFT for multiplexed protein analysis in FFPE tissues, we stained protein ILF3, Nucleophosmin, and hnRNP K through reiterative immunofluorescence cycles on a human FFPE tonsil tissue (Figure 8A). We also stained these three proteins by the conventional TSA method (Figure 8B). The staining patterns and signal intensities obtained by the two approaches are consistent with each other (Figure 8D). These results indicate that multiplexed in situ protein profiling can be achieved in FFPE tissues using the high-performance CFT.

The existing reiterative protein staining approaches [7,8,9,10,11,12] require the proteins to be profiled in the order of increasing abundances, so that the leftover signals produced in the previous cycles may not interfere with the protein quantification in the following cycles. However, sometimes the amount of the precious clinical and biological samples is limited, and thus it can be impossible to have knowledge of the relative protein expression levels in advance. Additionally, the order of the protein abundances in the varied cell types and states in the same specimen can be different. As a result, it might not be feasible to have an ideal protein analysis sequence that works for all the distinct cell types in the same specimen. Due to its high signal removal efficiency, our CFT approach may partially address those issues by eliminating the requirement of knowing the relative protein abundances in advance. To assess whether proteins can be profiled in the order of decreasing expression levels, we stained protein hnRNP K, nucleophosmin and ILF3 sequentially on a human FFPE tonsil tissue (Figure 8C). The generated staining patterns and signal intensities of all three proteins closely resembled the ones obtained by the conventional TSA approach (Figure 8D). These results suggest that the high signal removal efficiency of our approach enables it to partially eliminate the requirement of the prior knowledge of protein abundances and potentially allow accurate protein quantification regardless of the protein profiling order.

## 3. Materials and Methods

### 3.1. General Information

Chemicals and solvents were purchased from Sigma-Aldrich (St. Louis, MO, USA), and were used directly without further purification. Bioreagents were purchased from Invitrogen (Waltham, MA, USA), unless otherwise noted.

### 3.2. Synthesis of Tyramide-N_3_-Cy5

Cy5-N_3_ acid (8.3 μmol), prepared according to the literature [18], *N,N,N*′*,N*′-Tetramethyl-*O*-(*N*-succinimidyl)uronium tetrafluoroborate (TSTU) (12.5 mg, 41.5 μmol) and *N,N*-diisopropylethylamine (DIPEA) (7.3 μL, 41.5 μmol) were mixed in anhydrous DMF (300 μL) and stirred at room temperature. The complete transformation of Cy5-N_3_ acid into Cy5-N_3_ NHS ester was observed via TLC (DCM:methanol = 5:1) after 30 min. Then, tyramine hydrochloride (6.23 mg, 41.5 μmol) and DIPEA (14.6 μL, 83.0 μmol) were added into the reaction mixture and stirred for 2 h. The solvent was evaporated, and the solid residue was purified by preparative TLC (DCM:methanol = 5:1) to obtain the product as a dark blue solid. Tyramide-N_3_-Cy5 was further purified by semipreparative reversed phase HPLC (HPLC gradient: A, 100% 0.1 M TEAA; B 100% MeCN; 0–2 min, 5% B (flow 2–5 mL/min); 2–10 min, 5–22% B (flow 5 mL/min); 10–15 min, 22–30% B (flow 5 mL/min); 15–20 min, 30–40% B (flow 5 mL/min); 20–25 min, 40–50% B (flow 5 mL/min); 25–30 min, 50–60% B (flow 5 mL/min); 30–32 min, 60–70% B (flow 5 mL/min); 32–35 min, 70–95% B (flow 5 mL/min); 35–37 min, 95% B (flow 5 mL/min); 37–39 min, 95–5% B, (flow 5 mL/min); 39–42 min, 5% B (flow 5–2 mL/min)). The fraction with retention time of 26.4 min was collected. After evaporating all the solvents, the residue was coevaporated with methanol twice to obtain pure tyramide-N_3_-Cy5 as a blue solid (3.53 mg, 3.10 μmol). ^1^H NMR (500 MHz MeOD): 8.25 (2H, SO_3_H, t, J = 16.5 Hz), 7.90 (4H, Ar-H, m), 7.35–7.10 (5H, Ar-H, m), 6.95 (1H, Ar-H, m), 6.9 (2H, Ar-H, d, J = 10.5 Hz), 6.62–6.52 (3H, 2 × Ar-H and CH=, m), 6.27–6.14 (2H, CH=, dd, J = 17 Hz, J = 8.0 Hz), 4.7 (1H, CH-N_3_, t, J = 8.0 Hz), 4.10–3.96 (4H, 2 × CH_2_N, m), 3.90 (2H, OCH_2_, t, J = 9.0 Hz), 3.84–3.77 (3H, OCH_2_, Hb and CH_2_C(O), m), 3.65–3.60 (1H, OCH_2_, Ha, m), 3.53 (2H, CH_2_N, t, J = 7.5 Hz), 3.37 (2H, CH_2_N, t, J = 5.0 Hz), 3.29 (2H, CH_2_O, t, J = 5.0 Hz), 2.57 (2H, CH_2_N, t, J = 9.0 Hz), 2.13–2.01 (4H, CH_2_ and CH_2_C(O), m), 1.65–1.52 (15H, 4 × CH_3_ and CH3, m), 1.33–1.24 (6H, 3 × CH_2_, m). HRMS (ESI-, *m*/*z*) calculated for C_57_H_71_N_8_O_13_S_2_ ((M − 2H)^−^): 1137.4529, found: 1137.4327.

### 3.3. Deparaffinization and Antigen Retrieval of FFPE Tonsil Tissue

An FFPE tonsil tissue (Novus Biologicals, Littleton, CO, USA, NBP2-30207) was heated at 60 °C for 1 h before being deparaffinized in xylene 5 times, each for 4 min. Afterwards, the slide was immersed in 100% ethanol for 4 min twice, 95% ethanol for 4 min, 75% ethanol for 4 min then rinsed with DI water. Heat induced antigen retrieval (HIAR) was performed on the slide by using the microwave (Fisher Scientific, Waltham, MA, USA 03-391-462). The slide was immersed in 100 mL antigen retrieval buffer (diluted 100 times from Abcam 100× Citrate Buffer pH 6.0 with DI water) and heated in the microwave for 2 min and 40 s at level 10 setting and then for 14 min at level 2 setting. After cooling down for 20 min, the slide was rinsed with DI water.

### 3.4. Protein Staining in FFPE Tonsil Tissue

To deactivate the endogenous peroxidase, the slide was incubated with 3% H_2_O_2_ for 10 min at room temperature, followed by washing 3 times using 1× PBT (0.1% (*vol*/*vol*) Triton X-100 in 1× PBS). Next, the slide was incubated with 1× blocking buffer (1% (*wt*/*vol*) bovine serum albumin, 0.1% (*vol*/*vol*) Triton X-100, 10% (*vol*/*vol*) normal goat serum) for 30 min at room temperature. Then, the slide was incubated with 5 μg/mL rabbit anti hnRNP K, HRP (Abcam, Cambridge, MA, USA, ab204456) in blocking buffer for 1 h, and washed 3 times with 1× PBT, each for 5 min. The slide was stained with tyramide-N_3_-Cy5 at the concentration of 10 nmol/mL in amplification buffer for 7 min at room temperature, then washed 3 times with 1× PBT, each for 5 min. The stained tissue was incubated with GLOX buffer (10 mM Tris HCl and 0.4% glucose in 2× saline-sodium citrate (SSC) buffer (30 mM trisodium citrate, 300 mM sodium chloride, pH = 7.0)) at room temperature for 1 min, and subsequently imaged in GLOX solution (1% catalase and 0.37 mg/mL glucose oxidase in GLOX buffer).

### 3.5. Fluorophore Cleavage and HRP Deactivation

The stained tissue was incubated with 100 mM 1,3,5-Triaza-7-phosphaadamantane (PTA) and 100 mM tris(2-carboxyethyl)phosphine (TCEP) sequentially at 40 °C, each for 30 min. After 5 min wash with PBT and 1× PBS, each for 3 times, the tissue was imaged in GLOX solution.

### 3.6. Effect of Cleavage by PTA

After being deparaffined and the antigen retrieved, the FFPE tonsil tissue was incubated with 100 mM PTA overnight at 40 °C. Then, the slide was washed 3 times with 1× PBT, each time for 5 min. Subsequently, the slide was incubated with 5 μg/mL rabbit anti ILF3, HRP (Abcam; ab206250) and stained with tyramide-N_3_-Cy5. In the control experiment, without the PTA treatment in advance, another FFPE tonsil tissue was directly stained with rabbit anti ILF3, HRP (Abcam; ab206250) and tyramide-N_3_-Cy5.

### 3.7. Cell Culture

HeLa CCL-2 cells (ATCC) were maintained in a humidified atmosphere with 5% CO_2_ at 37 °C in Dulbelcco’s modified Eagle’s Medium (DMEM), which was supplemented with 100 U/mL penicillin, 100 g/mL streptomycin and 10% fetal bovine serum. Cells plated on chambered coverglass (200 μL medium/chamber) (Thermo Fisher Scientific) were allowed to reach ~60% confluency in 1 to 2 days.

### 3.8. Cell Fixation and Permeabilization

Cultured HeLa cells were fixed with 4% formaldehyde (Polysciences) in 1× phosphate buffered saline (PBS) (pH = 7.4) for 15 min at 37 °C. Subsequently, the cells were washed with 1× PBS three times, each for 5 min. After being permeabilized with PBT (0.1% Triton-X 100 in 1× PBS) at room temperature for 10 min, cells were washed with 1× PBS three times, each for 5 min.

### 3.9. Immunofluorescence with CFT

To block endogenous peroxidase, fixed and permeabilized HeLa cells were incubated with 0.15% H_2_O_2_ in PBT for 10 min, and subsequently washed three times with 1× PBS, each for 5 min. Then, the cells were blocked with 1× blocking buffer (0.1% (*vol*/*vol*) Triton X-100, 1% (*wt*/*vol*) bovine serum albumin and 10% (*vol*/*vol*) normal goat serum) for 1 h at room temperature. Subsequently, the cells were incubated with 5 μg/mL HRP conjugated primary antibodies in 1× blocking buffer for 1 h, followed by washing 3 times with PBT, each time for 5 min. Then, the cells were incubated with tyramide-N_3_-Cy5 at the concentration of 10 nmol/mL in amplification buffer (0.1 M Boric acid, pH = 8.5) for 7 min. Afterwards, the cells were washed quickly with PBT twice, and then washed with PBT again 3 times, each for 5 min. The stained cells were incubated with GLOX buffer (10 mM Tris HCl and 0.4% glucose in 2× saline-sodium citrate (SSC) buffer (30 mM trisodium citrate, 300 mM sodium chloride, pH = 7.0)) at room temperature for 1 min, and subsequently imaged in GLOX solution (1% catalase and 0.37 mg/mL glucose oxidase in GLOX buffer). The primary antibodies used in this work include rabbit anti-HMGB1, HRP (Thermo Fisher Scientific; PA5-22722), rabbit anti-HDAC2, HRP (Abcam; ab195851), rabbit anti-TDP43, HRP (Abcam; ab193850), rabbit anti-PABPN1, HRP (Abcam; ab207515), rabbit anti-hnRNP A1, HRP (Abcam; ab198535), mouse anti-Nucleolin, HRP (Abcam; ab198492), rabbit anti-Histone H4 (acetyl K16), HRP (Abcam; ab200859), mouse anti-hnRNP K, HRP (Abcam; ab204456), rabbit anti-ILF3, HRP (Abcam; ab206250) and mouse anti-Nucleophosmin, HRP (Abcam; ab202579).

### 3.10. Multiplexed Protein Imaging in Cells

Fixed and blocked HeLa cells were incubated with HRP conjugated antibodies at the concentration of 5 μg/mL for 1 h at room temperature, and subsequently stained with tyramide-N_3_-Cy5. Afterwards, the stained cells were imaged and then incubated with 100 mM PTA and 100 mM TCEP sequentially at 40 °C, each for 30 min. The cells were imaged again, followed by the next cycle of immunofluorescence. The sequentially applied antibodies include rabbit anti-HMGB1, HRP (Thermo Fisher Scientific; PA5-22722), rabbit anti-HDAC2, HRP (Abcam; ab195851), rabbit anti-TDP43, HRP (Abcam; ab193850), rabbit anti-PABPN1, HRP (Abcam; ab207515), rabbit anti-hnRNP A1, HRP (Abcam; ab198535), mouse anti-Nucleolin, HRP (Abcam; ab198492), rabbit anti-Histone H4, HRP (acetyl K16) (Abcam; ab200859), mouse anti-hnRNP K, HRP (Abcam; ab204456), rabbit anti-ILF3, HRP (Abcam; ab206250) and mouse anti-Nucleophosmin, HRP (Abcam; ab202579). In control experiments, fixed and blocked HeLa cells were incubated with HRP conjugated primary antibodies at the concentration of 5 μg/mL for 1 h at room temperature, and subsequently stained with Cy5-tyramide (PerkinElmer, Waltham, MA, USA).

### 3.11. Multiplexed Protein Imaging in FFPE Tonsil Tissue

After deparaffinization and antigen retrieval, the endogenous peroxidase in FFPE tonsil tissues was blocked by 3% H_2_O_2_. Subsequently, the slide was incubated with HRP conjugated antibodies at the concentration of 5 μg/mL for 1 h at room temperature, and then stained with tyramide-N_3_-Cy5. Afterwards, the stained tissues were imaged and then incubated with 100 mM PTA and 100 mM TCEP sequentially at 40 °C, each for 30 min. The tissues were imaged again, followed by the next cycle of immunofluorescence. The antibodies, rabbit anti-ILF3, HRP (Abcam; ab206250), mouse anti-Nucleophosmin, HRP (Abcam; ab202579) and mouse anti-hnRNP K, HRP (Abcam; ab204456), were applied in the forward and reverse orders on two different slides.

### 3.12. Imaging and Data Analysis

The HeLa cells and FFPE tonsil tissues were imaged using a Nikon Ti-E epifluorescence microscope with a 20× objective and a CoolSNAP HQ2 camera. C-FL DAPI HC HISN Zero Shift filter and Chroma filter 49,009 were used to image DAPI and Cy5, respectively. To image the same region of interest from different cycles, a motorized stage is used so that the coordination of the image area on the microscope stage is recorded and the motorized stage can automatically move to the same imaging area precisely in different cycles. Image analysis was performed with NIS-Elements Imaging software.

## 4. Conclusions

In summary, we have designed and synthesized a high performance CFT, and demonstrated that it can be applied to multiplexed, in situ protein profiling in culture cells and FFPE tissues. Compared to the existing multiplexed protein imaging technologies, our approach has the following advantages. First, with the signal amplification by HRP, our approach has dramatically improved detection sensitivity. As a result, it enables the precise quantification of low-expression proteins in highly autofluorescent specimens, and also significantly enhance the sample throughput by reducing the imaging time. Second, as the commercially available and well-validated antibodies are directly used in our approach, the time-consuming and expensive antibody-tag conjugation process is avoided. Third, by eliminating the carbamate group in the chemical structure of the high-performance CFT, the potential side reactions during CFT storage and protein staining are minimized. Finally, with the enhanced signal removal efficiency under a mild condition, our approach could allow multiple proteins to be precisely quantified regardless of the protein analysis order.

The multiplexing capacity of our approach depends on the number of reiterative analysis cycles and the number of proteins profiled in each cycle. We have shown that the protein antigenicity is preserved after the PTA overnight incubation, and we also documented previously that the integrity of the epitopes is maintained following the TCEP treatment for 24 h [8]. As each signal removal reaction only requires 30 min of the PTA and TCEP incubation, we envision that at least 20 to 30 reiterative cycles can be performed on the same sample. In each analysis cycle, after staining the different protein targets using CFT with varied fluorophores, the HRP can be deactivated [50] or the antibodies can be stripped [51] using the established methods. In this way, four or five different proteins can be quantified in each cycle. As a result, we anticipate that our approach has the potential to enable ~100 proteins to be profiled in the same specimen.

The high-performance CFT developed here can also be applied for in situ analysis of nucleic acids [52] and metabolics [53]. By performing these applications together with protein analysis, the combined DNA, RNA, protein and metabolic analysis can be achieved in single cells in their natural spatial contexts. Additionally, a program-controlled microfluidic device and a fluorescence microscope can be easily integrated to make an automatic highly multiplexed molecular imaging system. This comprehensive in situ molecular profiling platform will bring new insights into systems biology, biomedical research and precision medicine.

## Data Availability

The data presented in this study are available in article.

## Sample Availability

Samples of the compounds are available from the authors.
